# First report of *Meloidoyne enterolobii* infecting *Lagerstroemia indica* in Alabama, United States

**DOI:** 10.2478/jofnem-2025-0057

**Published:** 2025-12-07

**Authors:** Bisho Ram Lawaju, Jeremy Pickens, Kassie Conner, Weimin Ye, Kathy S. Lawrence

**Affiliations:** Department of Entomology and Plant Pathology, Auburn University, Auburn, AL 36849; Alabama Cooperative Extension System, Mobile, AL 36689; Nematode Assay Section, North Carolina Department of Agriculture and Consumer Services, Raleigh, NC 27607

**Keywords:** Alabama, crape myrtle, diagnosis, *Lagerstroemia indica*, *Meloidogyne enterolobii*, root-knot nematode

## Abstract

Crape myrtle (*Lagerstroemia indica*), a popular ornamental tree in the southern United States, was observed with severe root galls in a commercial nursery in central Alabama. Soil samples yielded high populations of root-knot nematode juveniles (104–277 J2/100 cm^3^). Morphology and morphometrics were consistent with *Meloidogyne* spp., but species-specific PCR for *M. incognita*, *M. arenaria*, *M. javanica*, and *M. hapla* were all negative. Sequencing of 18S, 28S rDNA, and mitochondrial COII-16S rRNA regions showed >99.5% identity to *M. enterolobii*. Two *M. enterolobii*-specific primers confirmed the diagnosis. A clonal population was maintained on tomatoes and used in a host range and pathogenicity assay. High reproduction (reproduction factor ≥8.6) occurred on pepper, sweet potato, tomato, and watermelon, while limited reproduction was found on peanut, corn, and one cotton cultivar. This is the first report of *M. enterolobii* infecting a commercial plant in Alabama. Due to its aggressive nature, wide host range, and ability to overcome resistance, there is an urgent need for monitoring and management strategies against this species.

Crape myrtle (*Lagerstroemia indica*) is a widely grown ornamental tree valued for its vibrant flowers and adaptability in subtropical and tropical climates, especially in the southern United States. On a commercial nursery farm in central Alabama, severe root galls exceeding 20 mm in diameter enveloping the entire root system were observed from 0.5% of the crape myrtle “Natchez” plants in February 2025. Soil samples collected from the affected field contained second-stage juveniles (J2) of root-knot nematodes (*Meloidogyne* spp.), with population densities ranging from 104 to 277 J2 per 100 cm^3^ of soil. Morphometric analysis of J2 individuals (*n* = 20) revealed measurements consistent with *Meloidogyne* spp.: body length = 413.9 ± 27.6 (352.0–462.0) μm; body width = 15.2 ± 1.62 (9.84–17.22) μm; stylet length = 14.4 ± 1.54 (12.3–17.2) μm; esophageal length = 115.8 ± 8.15 (98.4–127.9) μm; tail length = 64.5 ± 7.58 (46.7–78.7) μm; and anal body width = 12.5 ± 1.75 (7.38–14.76) μm. For species identification, total DNA was extracted from individual J2 (*n* = 15) and female (*n* = 5) nematodes and initially tested using species-specific primers: IncK14F/IncK14R (*M. incognita*-specific) (Randig et al., 2002), Far/Rar (*M. arenaria*-specific), Fjav/Rjav (*M. javanica*-specific) (Zijlstra et al., 2000), and MH0F/MH1R (*M. hapla*-specific) (Williamson et al., 1997). These assays did not yield positive amplification. Subsequently, PCR amplification was performed using universal primers targeting the 18S rDNA (18S965/18S1573R and SSUF07/SSUR26), 28S D2-D3 region (D2A/D3B) (Ye et al., 2015), and the mitochondrial cytochrome oxidase subunit II (COII)-16S rRNA region (C2F3/1108) (Powers and Harris, 1993). The resulting amplicons were sequenced and compared with sequences in the NCBI GenBank database. The 18S rDNA sequence (GenBank accession nos. PV984042 and PV984044) were 100% identical to several *M. enterolobii* sequences but also shared >99.5% similarity to sequences of *M. incognita*, *M. arenaria*, and *M. javanica.* The 28S rDNA fragment (PV987946) was 100% identical to *M. enterolobii* sequences reported from different regions of the world, whereas the COII-16S mitochondrial fragment (PX514999) showed 99.87% identity (1-bp difference) with 24 *M. enterolobii* sequences from diverse global geographic origins. To further confirm the species identity, additional PCR assays were conducted using two *M. enterolobii*-specific primer sets, MeF/MeR (Long et al., 2006) and MK7F/MK7R (Tigano et al., 2010). The MeF/MeR primers amplified DNA from all samples tested (*n* = 30), whereas MK7F/MK7R amplified 70% of the samples. These results confirmed the identity of *M. enterolobii* infecting crape myrtle (*L. indica*) roots in Alabama.

To fulfill Koch's postulates and assess the host range and pathogenicity, a clonal population of *Meloidogyne enterolobii* was derived from a single-egg-mass culture. An individual egg mass was handpicked from an infected crape myrtle (*L. indica* “Natchez”) root system and inoculated onto tomato (*Solanum lycopersicum* “Rutgers”) maintained under greenhouse conditions. The nematode population originating from this single egg mass was subsequently propagated and maintained on fresh “Rutgers” tomato plants to provide inoculum for further experiments. Sixty days after inoculation, eggs were extracted from the infected roots and used to inoculate a host range test. Each 15-cm-diameter pot (500 cm^3^) filled with a 1:2 mix of pasteurized river sand and sandy loam soil was infested with 5,000 eggs. Test hosts included tomato (*S. lycoperscicum* “Rutgers”), peanut (*Arachis hypogaea* “GA06” and “AU17”), cotton (*Gossypium hirsutum* “DP 2141NR B3XF” and “PHY 340 W3FE”), watermelon (*Citrullus lanatus* “Charleston Grey”), pepper (*Capsicum annuum* “California Wonder”), tobacco (*Nicotiana tabacum* “NC 95”), alongside corn (*Zea mays* “DKC68-95”), soybean (*Glycine max* “AG46XF2”), sweet potato (*Ipomoea batatas* “Beauregard”), and crape myrtle (*L. indica* “Natchez”). Each treatment had five replications and was maintained in a greenhouse at 25 ± 2°C in a completely randomized design. After 45 days, root systems were evaluated for galling and nematode reproduction. Nematode eggs were extracted from the roots using 0.6% NaOCl (Hussey and Barker, 1973) followed by washing and centrifuging in a molar sucrose solution. Reproduction factor (RF = final egg count/initial egg count, based on five replicates) varied across hosts. The RF were <1 indicating limited reproduction occurred on peanut (RF = 0.2 ± 0.13 and 0.6 ± 0.21), cotton “DP 2141NR B3XF” (RF = 0.8 ± 0.32), and corn (RF = 0.3 ± 0.27). High reproduction was observed on pepper (RF = 55.7 ± 27.72), sweet potato (RF = 18.5 ± 8.92), tomato (RF = 14.4 ± 8.26), watermelon (RF = 8.6 ± 3.85), tobacco (RF = 2.4 ± 1.31), crape myrtle (RF = 1.9 ± 0.52), soybean (RF = 1.4 ± 0.33), and cotton “PHY 340 W3FE” (RF = 1.2 ± 0.58). These results were consistent with previously reported host ranges for *M. enterolobii* and *M. incognita* race 4 (Yang and Eisenback, 1983; Hartman and Sasser, 1985; Rutter et al., 2019). To our knowledge, this is the first report of *M. enterolobii* infecting a commercial plant in Alabama. Due to its aggressive nature, ability to overcome root-knot nematode resistance genes, broad host range, and limited management options (Kiewnick et al., 2009; Rutter et al., 2019), *M. enterolobii* represents a serious threat to ornamental and agricultural crops in the region. This finding highlights the urgent need for surveillance and implementation of strategies to prevent its spread in major crop production systems in the state.
